# Lipid species profiling of bronchoalveolar lavage fluid cells of horses housed on two different bedding materials

**DOI:** 10.1038/s41598-023-49032-1

**Published:** 2023-12-08

**Authors:** Jenni Mönki, Minna Holopainen, Hanna Ruhanen, Ninja Karikoski, Reijo Käkelä, Anna Mykkänen

**Affiliations:** 1 Department of Equine and Small Animal Medicine, Faculty of Veterinary Medicine, University of Helsinki, Viikintie 49, P.O. Box 57, 00014 Helsinki, Finland; 2 Helsinki University Lipidomics Unit (HiLIPID), Helsinki Institute of Life Science (HiLIFE), and Biocenter Finland, University of Helsinki, Biocenter 3 Viikinkaari 1, P.O. Box 65, 00014 Helsinki, Finland; 3 Molecular and Integrative Biosciences Research Programme, Faculty of Biological and Environmental Sciences, University of Helsinki, Viikinkaari 1, P.O. Box 65, 00014 Helsinki, Finland

**Keywords:** Pathogenesis, Respiratory tract diseases

## Abstract

The lipidome of equine BALF cells has not been described. The objectives of this prospective repeated-measures study were to explore the BALF cells’ lipidome in horses and to identify lipids associated with progression or resolution of airway inflammation. BALF cells from 22 horses exposed to two bedding materials (Peat 1—Wood shavings [WS]—Peat 2) were studied by liquid chromatography-tandem mass spectrometry (LC–MS/MS). The effects of bedding on lipid class and species compositions were tested with rmANOVA. Correlations between lipids and cell counts were examined. The BALF cells’ lipidome showed bedding-related differences for molar percentage (mol%) of 60 species. Whole phosphatidylcholine (PC) class and its species PC 32:0 (main molecular species 16:0_16:0) had higher mol% after Peat 2 compared with WS. Phosphatidylinositol 38:4 (main molecular species 18:0_20:4) was higher after WS compared with both peat periods. BALF cell count correlated positively with mol% of the lipid classes phosphatidylserine, sphingomyelin, ceramide, hexosylceramide, and triacylglycerol but negatively with PC. BALF cell count correlated positively with phosphatidylinositol 38:4 mol%. In conclusion, equine BALF cells’ lipid profiles explored with MS-based lipidomics indicated subclinical inflammatory changes after WS. Inflammatory reactions in the cellular lipid species composition were detected although cytological responses indicating inflammation were weak.

## Introduction

Equine airways are sensitive to airborne irritants, and traditional stabling can cause airway inflammation in healthy horses^1^. Equine asthma (EA) is a prevalent, chronic lower airway inflammatory disease of adult horses^2,3^. As exposure to a myriad of inhaled particles is important in EA aetiology and exacerbation, EA is a frequent problem in circumstances where horses are kept indoors for long^4^. Current consensus divides EA syndrome into mild-moderate (mild-moderate equine asthma; MEA) and severe (severe equine asthma; SEA) phenotypes. MEA encompasses the disease that was formerly known as inflammatory airway disease (IAD), whereas SEA is equivalent to the previously used term recurrent airway obstruction (RAO)^5^.

In practice, inflammatory airway diseases of horses are commonly diagnosed by history, clinical signs, and bronchoalveolar lavage fluid (BALF) cytology^6^, which can be complemented with dynamic respiratory tests^7^. However, cytology is limited in assessing inflammation in equine lower airways and has some limitations as a diagnostic tool. Normal variation in BALF neutrophil percentage, both inter- and intraindividually, is remarkable and can be affected by many factors^8–17^. Monitoring inflammation resolution is challenging, as neutrophils may reside in the lungs even for weeks despite clinical remission, which makes interpretation of BALF cytology difficult^18^. Therefore, the cut-off values for normal neutrophil percentages in equine BALF have been debated^5,18–20^. Asthma subtypes have been identified in humans but are poorly characterized in horses^5^. These issues emphasize the need for new diagnostic tools to detect equine lower airway inflammation.

Metabolomic analysis techniques have been used to identify new biomarkers of airway inflammation^21–23^. Lipidomics is a subfield of metabolomics that has not been widely explored in equine medicine^24–34^. Lipids have multiple cellular functions, such as membrane structural components, signalling molecules, membrane protein recruitment platforms, ligands that modulate protein functions, and energy sources^35,36^. As many proinflammatory and proresolving mediators are lipids, lipidomics is a promising method to study equine airway inflammation^37^. In human medicine, lipidomics has been used to investigate mechanisms and treatments of asthma and other lung diseases^38–44^. In horses, lipidomic studies have thus far focused on analysing plasma and surfactant samples from horses affected by different subtypes of EA^31,32^. Increased amounts of cyclic phosphatidic acid (cPA) and diacylglycerol (DAG) were detected in surfactant from severely asthmatic horses during exposure to hay^31^. Another study revealed that horses with SEA had altered surfactant phospholipid content and composition (increased ceramides, decreased phosphatidylglycerol, and increased cPA), whereas only minor changes were observed in horses with mild neutrophilic EA when compared with healthy individuals^32^. In the same study, the plasma lipidomic profile was significantly different in all groups of asthmatic horses compared with controls, and different asthma categories had different changes compared with each other^32^.

No previous lipidomic research has been conducted on equine BALF cells. Cellular membranes are the main source of the lipid mediators that act on proinflammatory, anti-inflammatory, and proresolving phases of the inflammatory cascade^36^, which makes BALF cells suitable to study from a lipidomics perspective. In the current study, subclinical inflammation in the airways of healthy horses was induced by switching bedding. BALF cells were then investigated with the aim of identifying individual lipids or their composition patterns that are associated with progression or resolution of airway inflammation. The bedding materials were selected based on a previous experiment in which peat caused less neutrophilic lower airway inflammation in horses compared with wood shavings^45^.

## Methods

### Ethical considerations

In animal handling and sample collection, the European Union recommendation directives (2010/63/EU) and national animal welfare and ethical legislation set by the Ministry of Agriculture and Forestry in Finland were followed. The experimental procedures were approved by the National Ethical Committee for Animal Experiments in Finland. The institutes that owned the horses also approved the study protocols.

### Sample size calculations

Sample size was calculated (https://homepage.univie.ac.at/robin.ristl/samplesize.php?test=pairedttest) with BALF neutrophil percentage as a primary outcome (2-tailed, power 0.8, significance 0.05, standard deviation 2, estimated difference in means 2, estimated group size 18 horses), which suggested that a sample > 18 horses was sufficient for this study.

### Animals and management

Twenty-two healthy riding school horses with no known history of chronic respiratory disease were used. The horses performed their usual routines and were fed haylage and pelleted compound feed (Krafft Grund, Lantmännen, Sweden).

### Sample collection and processing

**Figure 1:**
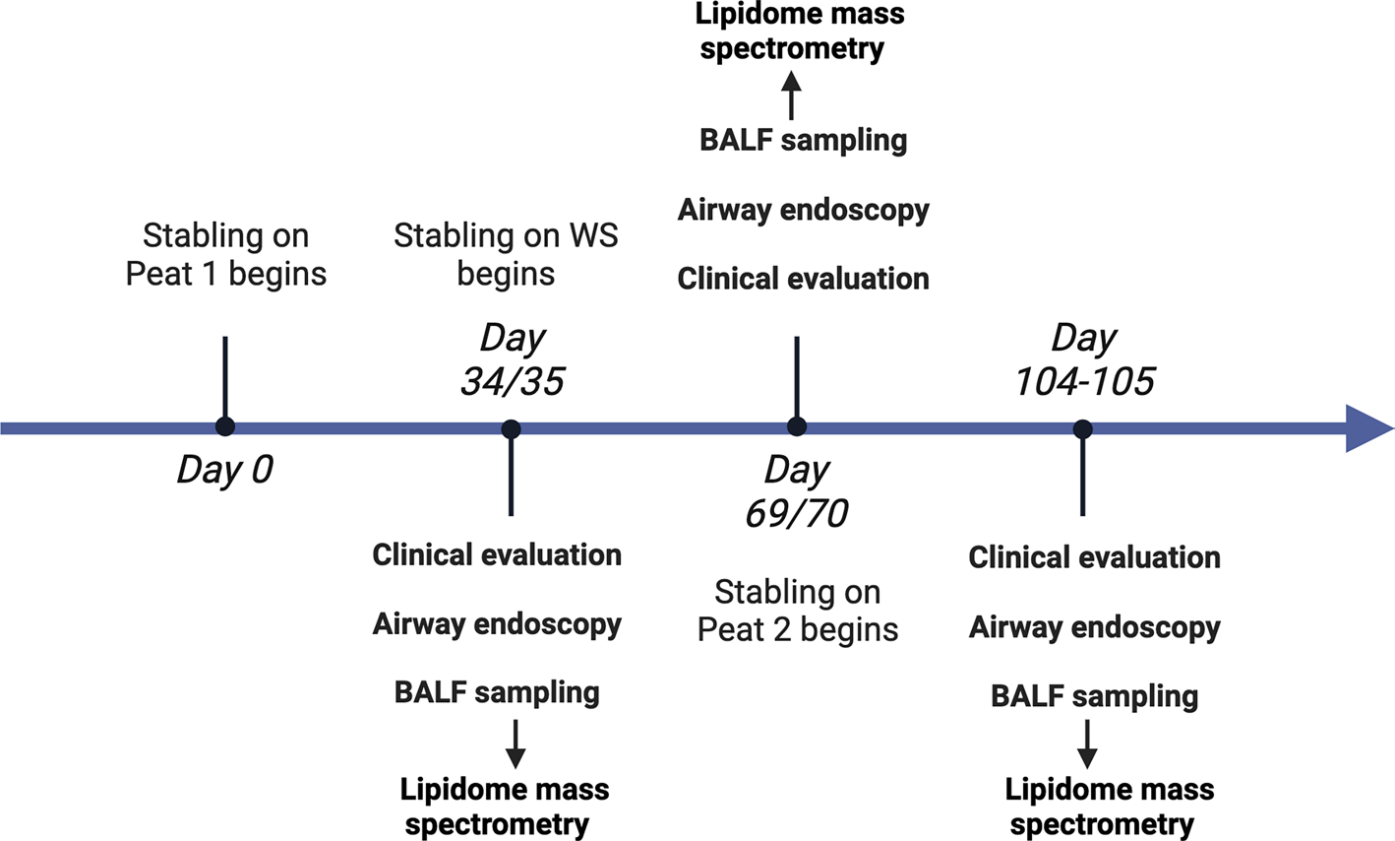
Timeline of the study design.

The experimental design is shown as a timeline in Fig. 1, and the details of the study protocol are described elsewhere^45^. In brief, the original study was performed using a repeated-measures study design comparing the effects of two bedding materials on equine lower airway inflammation. Horses acted as their own controls and peat was considered a reference bedding material. Each bedding material was used for all horses for 35 consecutive days in the following order: peat—wood shavings—peat (Fig. 1). Later in this manuscript, these periods are abbreviated as Peat 1, Wood shavings (WS), and Peat 2. Routine airway endoscopy, including scoring of tracheal mucus and cytological sampling (tracheal wash, BALF with blind technique), were performed on days 34 or 35 of each period^45^. Pooled BALF samples were filtered through a single-layer cotton gauze and fluid volume was recorded. BALF cell count was determined using trypan blue stain (1:1), after which the sample was cytocentrifuged (Thermo Scientific Cytospin 4 centrifuge; Thermo Fisher Scientific, Waltham, MA, USA). All slides were stained with May-Grünwald-Giemsa stain. A single blinded experienced observer counted 400 cells for BALF under light microscopy at 400× magnification, and performed differential counts for macrophages, lymphocytes, neutrophils, eosinophils, mast cells, and epithelial cells. The results were expressed as percentages of total cells. After the initial cytological analysis, different fractions of the BALF samples were stored at − 80 °C until further use. The cell fractions of the BALF were thawed, centrifuged at 700 ×*g* for 10 min at 4 °C, and supernatant was discarded immediately before lipid extraction of the cell pellets, including all leukocyte types of BALF.

### Lipidomics

Lipids were extracted from BALF cells according to Folch et al.^46^ and dissolved in chloroform/methanol 1:2 (by vol). An internal standard mixture containing phosphatidylcholine (PC) 14:1/14:1, PC 22:1/22:1, phosphatidylethanolamine (PE) 14:0/14:0, phosphatidylserine (PS) 14:0/14:0, ceramide (Cer) 18:1;O2/17:0, cholesterol ester (CE) 14:0, triacylglycerol (TG) 14:0/14:0/14:0, and TG 18:0/18:0/18:0 (Avanti Polar Lipids and Merck) was added and analysis of lipid-species composition was performed using an LC–MS/MS approach. Chromatographic separation was performed in gradient mode using an Agilent 1290 Infinity HPLC system equipped with a Luna Omega C18 100 Å (50 × 2.1 mm, 1.6 µm) column (Phenomenex) and employing an acetonitrile/water/isopropanol-based solvent system^47^ with flow rate of 0.200 mL/min and column temperature 25 °C. The column eluent was infused into the electrospray source of an Agilent 6490 Triple Quad LC/MS with iFunnel Technology and spectra were recorded using both positive and negative ionization modes. TGs and CEs were detected as (M + NH_4_)^+^ ions from MS + scan, Cers and hexosylceramides (HexCer) using sphingosine 18:1-specific precursor ion (P) scan of m/z 264 (P264), and phospholipids using head group specific P or neutral loss (NL) scans [PC and sphingomyelin (SM): P184, PE: NL141, phosphatidylinositol (PI): P241, and PS: NL87]. Mass spectra were processed using MassHunter Qualitative Navigator software (Agilent) and lipid species were quantified utilizing the internal and additional external standards (PI 16:0/16:0, SM 18:1;O2/17:0, glucosylceramide 18:1;O2/24:1, CE 19:0] and LIMSA software^48^. Lipid data are expressed as molar percentages (mol%) and lipid species are marked as follows: [sum of acyl chain carbons]: [sum of acyl chain double bonds] (e.g., PC 32:0 for species 16:0/16:0). Negative-mode precursor ion scans for acyl chains released from PE, PI, PS, and PC chloride adducts were used to confirm the fatty acyl combinations for the key lipids in this study.

An overview of the locations and functions of the main lipid classes that were studied are presented in Supplementary Table 1.

### Statistical analysis

Statistical analysis was performed with IBM SPSS Statistics for Windows, version 28.0.1, using repeated measures (rm)ANOVA with Bonferroni correction (to avoid false positives due to multiple comparisons) to compare the relative concentrations of the lipid classes and individual lipid species profiles of the horses from the three consecutive samplings (n = 22 for each). The post-hoc tests revealing pair-wise differences between each sampling used estimated marginal means. Ln transformation was used for BALF cell count and neutrophil percentage. Changes in lipid compositions, BALF cell count, neutrophil percentage, and macrophage percentage between the bedding periods were investigated. Greenhouse–Geisser test was used for within-subject effects. BALF cell count, neutrophil percentage, and macrophage percentage were compared with the relative concentrations of lipid classes and lipid species PC 32:0 and PI 38:4, with known functional connections to surfactant synthesis and lung inflammation^49–51^ using Spearman rank correlation. In all tests, *p* < 0.05 was considered statistically significant. To indicate differences in lipid profiles of BALF cells from different bedding periods, heatmaps using z-scores were created in MetaboAnalyst 5.0. (Xia Lab @ McGill). The lipid species mol% data were log-transformed and autoscaled to z-scores (each value was subtracted by the mean of the variable, followed by dividing by the standard deviation) before analysis^52^. The heatmaps of glycerophospholipid (GPL; including PC, PE, PI, and PS) profiles were shown using the top 25 variables with the largest separation power and clustering of the samples and variables. The individual PC species correlations in the mol% data of the horse BALF cells were demonstrated by Principal Component Analysis (PCA) biplot using Sirius 8.5 (Pattern Recognition Systems, Bergen, Norway).

## Results

### Animals

Twelve Finnhorses and ten Warmbloods (twelve were mares and ten geldings) were used. Mean age of the horses was 11.5 years (range 4–18). No horses showed signs of respiratory disease or infections.

### Lipidomic analyses

#### Differences in lipid class composition between the bedding periods

**Figure 2:**
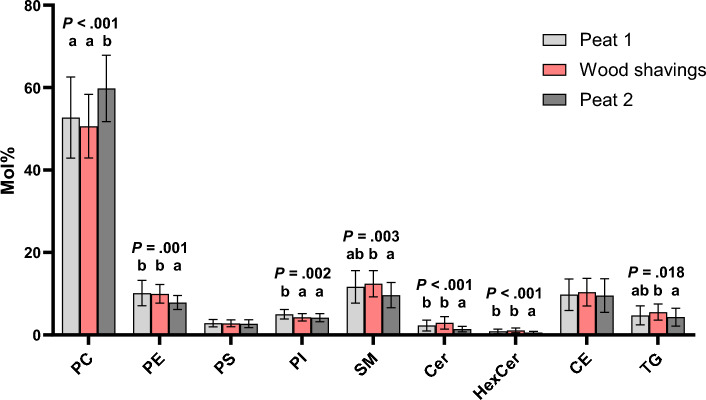
Total lipid class molar percentages (mol%) at three sampling points. Lipid classes with statistically significant differences between the bedding periods are indicated with letters addressing the groups in the order of the bars. The means with no common letter differ at the mentioned *p* level (rmANOVA followed by pairwise post-hoc test of means, N = 22).

The lipid class profiles of the BALF cells differed when the bedding periods were compared (Fig. 2). The statistically most significant (later briefly, significant) changes of class mol% were observed in PC, Cer, and HexCer (*p* < 0.001 for each). In PC, there was an increase from WS to Peat 2, which happened at the expense of several other lipid classes. The decreases were found in the proportions of PE, SM, Cer, HexCer, and TG (all significant, Fig. 2). In PI class mol%, there was a decrease from Peat 1 to both WS and Peat 2 (*p* = 0.002).

#### Differences in relative concentrations of individual lipid species between the bedding periods

**Figure 3:**
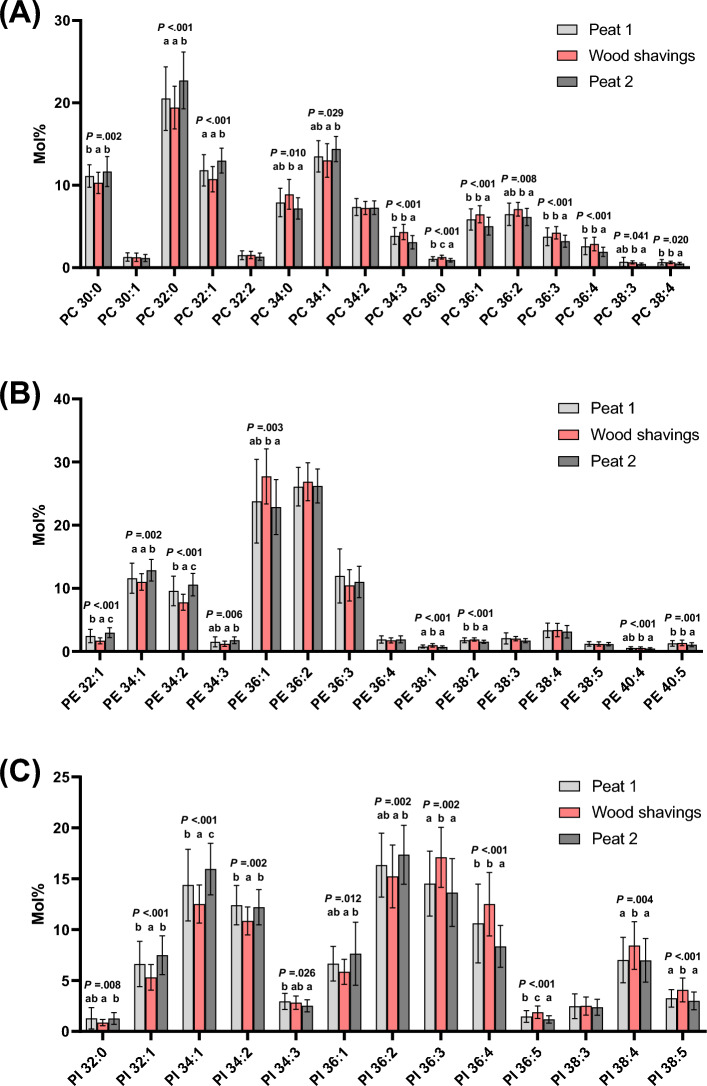
(**A**) Phosphatidylcholine (PC), (**B**) Phosphatidylethanolamine (PE), and (**C**) Phosphatidylinositol (PI) species molar percentages (mol%) at three sampling points. The lipid species with statistically significant differences between the bedding periods are indicated with letters addressing the groups in the order of the bars. The means with no common letter differ at the mentioned *p* level (rmANOVA followed by pairwise post hoc-test of means, N = 22).

**Figure 4:**
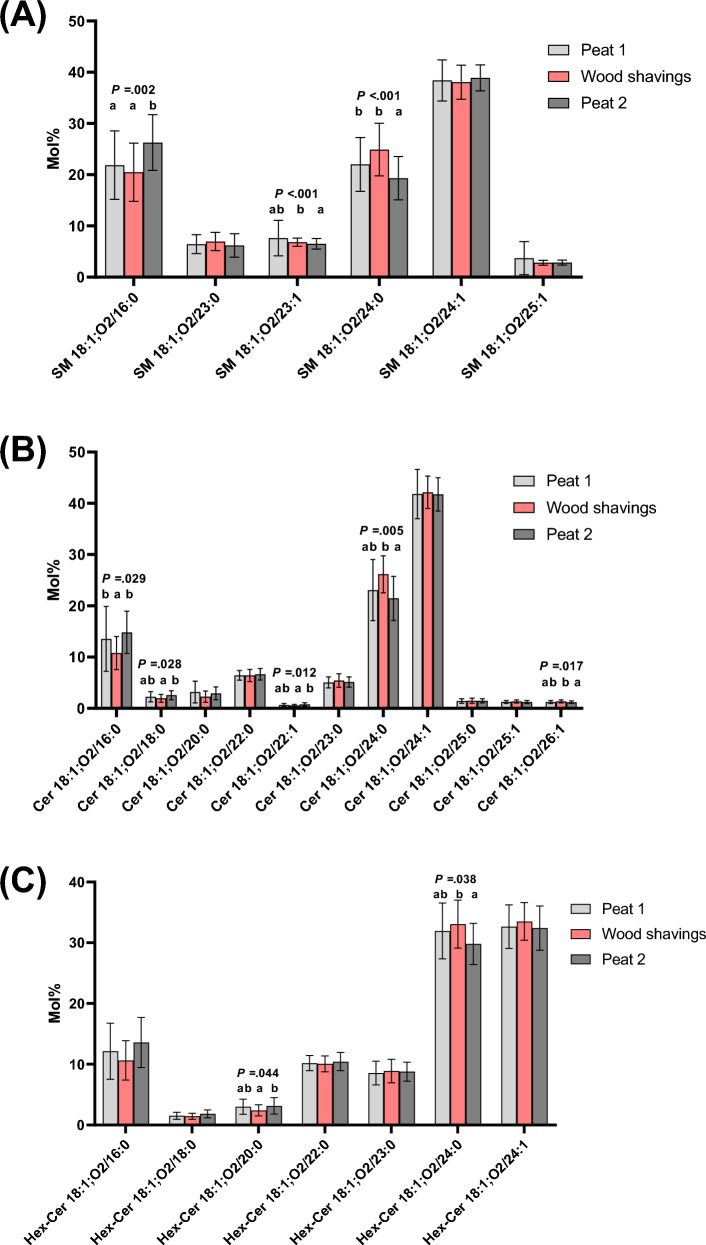
(**A**) Sphingomyelin (SM), (**B**) Ceramide (Cer), and (**C**) Hexosylceramide (HexCer) species molar percentages (mol%) at three sampling points. The lipid species with statistically significant differences between the bedding periods are indicated with letters addressing the groups in the order of the bars. The means with no common letter differ at the mentioned *p* level (rmANOVA followed by pairwise post-hoc test of means, N = 22).

Changes were observed between the bedding periods in 60 individual lipid species belonging to each lipid class (Figs. 3A–C, 4A–C, Table 2 Supplementary file). Most of the PC species exhibited a significant change (Fig. 3A). The trend was that the proportions of the shortest saturated and monounsaturated PC species increased from WS to the sampling after Peat 2. Simultaneously, the long chain species (also including the polyunsaturated species) decreased. Of special interest is the increase of the quantitatively most important PC 32:0 (main molecule 16:0_16:0), which was higher after Peat 2 than after Peat 1 and WS.

The change of PE species profile due to different bedding materials resembled the change in the PC profile by exhibiting increases from WS to Peat 2 sample for the species with the shortest acyl chains (PEs 32:1, 34:1, 34:2, and 34:3) (Fig. 3B). For the long-chain PE species (C36–40), the species that changed decreased from WS to Peat 2 sample. The PS profile changed only slightly (Supplementary Fig. 1A), with the increase of PS 34:1 for Peat 2 sampling the only significant change.

PI was another lipid class where most of the individual species changed when BALF cells of horses kept on different bedding were compared (Fig. 3C). The C32–34 PI species (except minor PI 34:3) increased from WS to Peat 2 sample. Among the C36 PI species, the mono- and diunsaturated species (PIs 36:1 and 36:2) increased from WS to Peat 2, but all the polyunsaturated C36–38 PI species (PIs 36:3, 36:4, 36:5, 38:4, and 38:5) had their highest proportions in the sample collected after WS. The WS values of these polyunsaturated PI species were in many cases higher than in the Peat 1 sample, and in all these species the values decreased for Peat 2 sampling.

The studied sphingolipid classes showed similar species profile changes (Fig. 4A–C). The molecular species 18:1;O2/16:0 had the lowest mol% in WS sample, different from Peat 2 in SM and from Peat 1 and 2 in Cer. The 18:1;O2/24:0 species decreased from WS to Peat 2 sample in SM, Cer, and HexCer, and few other class-specific changes in the minor species were detected.

In addition to the aforementioned alterations in the species profiles of membrane lipids, the composition of neutral storage lipids of the BALF cells changed due to the different beddings. In CE profile, the mol% of minor species CE 16:0 and CE 16:1 was lowest in the WS sample and increased significantly to Peat 2 sampling (Supplementary Fig. 1B). In TGs, 13 of the isobaric species had different mol% values after the different bedding periods. In general, the WS sample contained smaller proportions of the relatively short chain C45-48 TG species (carbon number is a sum from three acyl chains) than Peat 1 and Peat 2 samples. For most species in the range C50-53, WS values were higher than those of Peat 2 (Table 2, Supplementary file).

### Correlations between BALF cytology and lipidomic analyses

BALF cytology results of the three sampling points are shown in Table 1. The differences in BALF neutrophil and macrophage percentages between the bedding periods were investigated. Macrophage percentages were of interest, as alveolar macrophages are immunologically active cells that may participate in regulation of surfactant phospholipid concentrations^2,49,50^. BALF cell count differed between the bedding periods WS and Peat 2 (*p* = 0.046). The neutrophil percentage differed between the bedding periods (*p* = 0.037; pairwise tests: Peat 1 and Peat 2, *p* = 0.018; WS and Peat 2, *p* = 0.016). The macrophage percentage did not change between the bedding periods.

**Table 1:** Cytology results of BALF samples (N = 22) taken after each bedding period: the mean cell counts with SD ( ±) are presented as cells/µl with range in brackets; the mean percentages of each inflammatory cell population are shown (neutrophils, eosinophils, mast cells, macrophages, lymphocytes) with SD ( ±) and range in brackets.

	Cells/µl	Neutrophil %	Eosinophil %	Mast cell %	Macrophage %	Lymphocyte %
Peat 1	28.0 ± 15.8 (8–36)	2.4 ± 1.6 (0.5–6.3)	0.3 ± 1.3 (0–2.8)	3.8 ± 2.0 (0.5–7.5)	42.2 ± 9.8 (26–64.8)	51.1 ± 4.4 (29.5–69.8)
Wood shavings	29.4 ± 10.9 (15–61)	3.3 ± 3.4 (0.8–16.3)	0.2 ± 0.4 (0–1.8)	2.5 ± 1.3 (0–5.3)	42.3 ± 12.8 (27.8–59.3)	51.2 ± 12.5 (32.3–75.3)
Peat 2	21.7 ± 8.5 (13–86)	1.4 ± 0.8 (0–5.3)	0 ± 0 (0–0.3)	2.8 ± 2.3 (0–6)	47.0 ± 11.8 (27–63.3)	49.5 ± 13.8 (33–70)

BALF cell count correlated positively with PS (*r* = 0.268, *p* = 0.012), SM (*r* = 0.366, *p* < 0.001), Cer (*r* = 0.367, *p* < 0.001), HexCer (*r* = 0.374, *p* < 0.001), and TG (*r* = 0.429, *p* < 0.001) and negatively with PC (*r* = − 0.348, *p* < 0.001). As the mol% of PI 38:4 reacts sensitively to immunity stimulant in rodent macrophages^49^, and in this study the mol% of PI 38:4 of equine BALF cells increased after WS, we studied whether the BALF cell count or its inflammatory cell proportions correlated with the mol% of this specific lipid species. We observed that the BALF cell count correlated positively with PI 38:4 (*r* = 0.420*, p* < 0.001). In addition, since PC 32:0 (16:0_16:0) is the major phospholipid species in pulmonary surfactant, we additionally studied whether its mol% correlated with cell count or its inflammatory cell proportions. BALF cell count correlated negatively with PC 32:0 (*r* = − 0.225, *p* = 0.038). Neither neutrophil nor macrophage percentages correlated with PI 38:4 or PC 32:0. Neither did the studied sphingolipid (SM, Cer, HexCer) species with 16:0 acyl chain, which decreased during WS, correlate with the BALF cell count, neutrophil, or macrophage percentage.

BALF neutrophil percentage correlated negatively with CE (*r* = − 0.218, *p* = 0.044). BALF macrophage percentage correlated negatively with PC (*r* = − 0.336, *p* = 0.009) and Cer 18:1;O2/24:1 (*r* = − 0.305, *p* = 0.018) and positively with CE (*r* = 0.289, *p* = 0.025), TG (*r* = 0.352, *p* = 0.006), Cer (*r* = 0.279, *p* = 0.031), and HexCer (*r* = 0.255, *p* = 0.049).

### Differences in membrane lipid species composition between the bedding periods revealed by heatmap and principal component analyses

**Figure 5:**
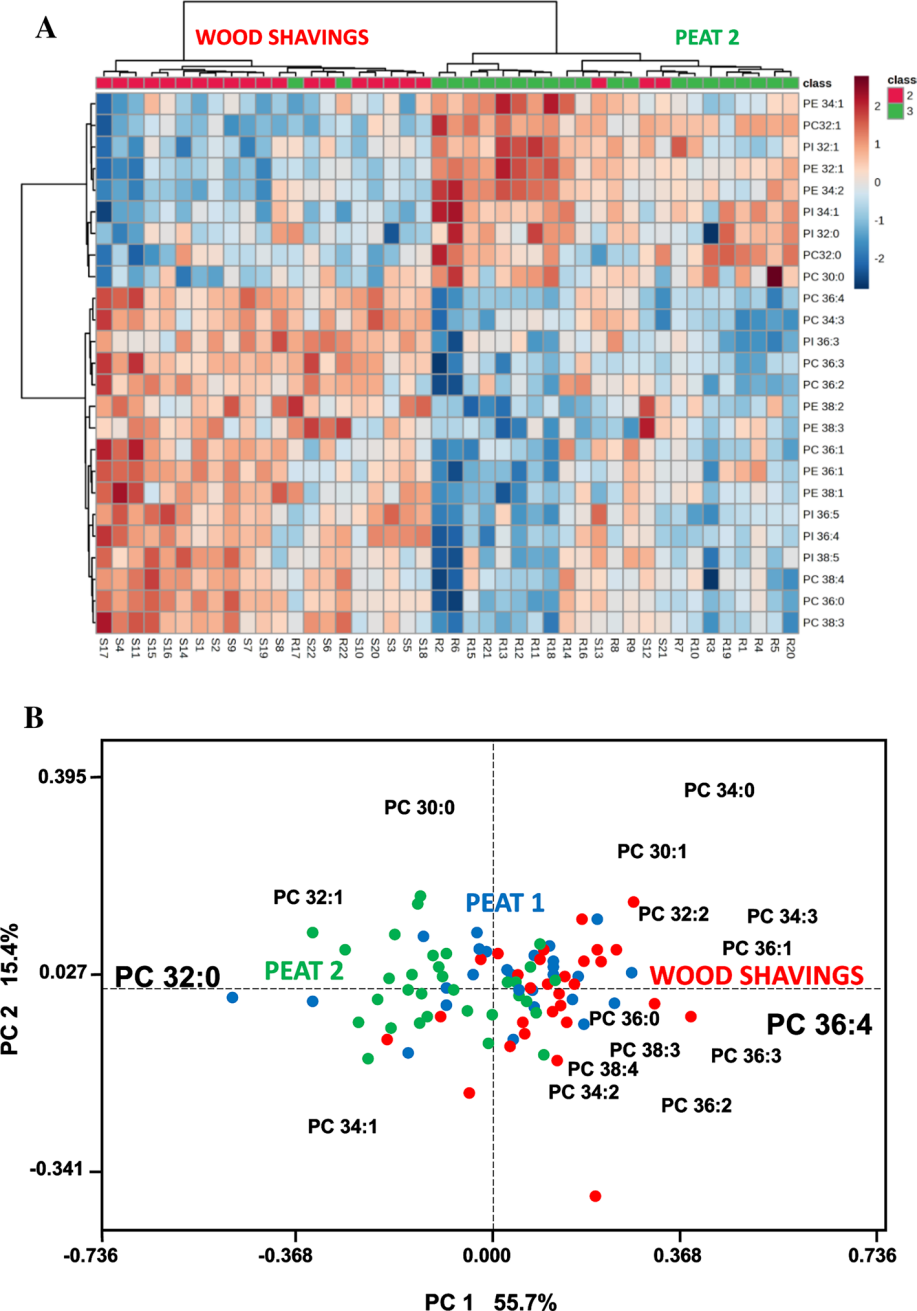
(**A**) Heatmap of the top 25 glycerophospholipid (GPL; including PC, PE, and PI) species with the largest separation power characterized the main difference in the membrane lipids between the WS (individuals marked SX) and Peat 2 (individuals marked RX) samples (N = 22). The GPL species were marked as follows: PC = phosphatidylcholine, PE = phosphatidylethanolamine, PI = phosphatidylinositol, and the numbers define the species by [sum of acyl carbons]:[sum of acyl double bonds]. (**B**) Principal Component Analysis biplot of BALF cell samples from horses sampled after each consecutive bedding (each color-coded circle represents one BALF cell sample) and using their PC species as loadings. Two Principal Components (PC1 and PC2) that together explain 71.1% of total data variation are used as the x- and y-axis. The PC 32:0 (16:0_16:0) high in the cell samples of the Peat 2 group correlated negatively with PC 36:4 (16:0_20:4) high in the cell samples of the Wood shavings group.

A heatmap (Fig. 5A) using the top 25 GPL species characterizes the main differences in the membrane lipids between WS and Peat 2 samples. The relatively short (C30–34) monounsaturated and saturated species of PC, PE, and PI were enriched in Peat 2 sample, whereas the WS sample was rich in the longer chain (largely C36–38) species and many of these long ones were polyunsaturated species. The heatmap clustered (based on similarity of the whole composition) 19 WS BALF cell samples correctly to the WS group and 3 to Peat 2 group. At the same time, 20 Peat 2 samples were clustered to Peat 2 group and 2 to WS group. The corresponding heatmap, including samples from the three bedding periods, is found in Supplementary Fig. 2.

In the PCA biplot (Fig. 5B), PC 32:0 (mainly with 16:0 chains) was located on the left with many peat period samples and most of the other saturated and monounsaturated PC species, whereas the polyunsaturated species were located on right with most WS samples. The polyunsaturated species included PC38:4 and PC 36:4, which isobaric species were found to contain arachidonic acid (20:4). Among the studied BALF cell samples, the negative correlation between PC 32:0 and PC 36:4 was especially strong (lines connecting these species to the plot origin formed an angle close to 180°).

## Discussion

To the best of our knowledge, this is the first study to describe the lipid profile of equine BALF cells using quantitative methods. In previous equine airway inflammation research, lipidomic analyses were performed from surfactant and plasma^31,32^. However, many more airway sample types have been used in human asthma lipidomics research. Differences in lipid composition between human asthmatics and controls have been found in plasma^53^, extracellular vesicles (EVs)^54^, BALF^55^, alveolar macrophages, bronchial epithelial cells and alveolar type II cells^56^ and sputum^57^.

The current study used BALF cells retrieved from healthy horses that, based on the lipidomics, had episodic subclinical inflammatory changes that subsequently resolved. Exposure to an unfavourable bedding material (WS) was associated with significant but minor elevations in BALF neutrophil percentages in a larger group of horses^45^. In this study, we found several differences between the bedding periods in the lipid class and individual lipid species profiles of the BALF cells (Figs. 2, 3, 4, Supplementary Fig. 1, and Supplementary Table 2). Some of the most interesting results were the increases of lipid species that contain 16:0 acyl chain (for example PC 32:0), which were apparent after Peat 2. The second intriguing result was that there was an increased mol% of PI 38:4 in BALF cells from horses that were housed on WS compared with both peat periods. The third main result was that in the sphingolipids (SM, Cer, HexCer) there were decreases of the 24:0 species after Peat 2, and increases of the 16:0 species in SM and Cer. Fourthly, the BALF cell count and BALF neutrophil percentages differed between the bedding periods. Most interestingly, there was a decrease after Peat 2 compared with WS in both parameters. When the BALF cytology and lipidomic analyses were compared, there were positive correlations between BALF cell counts with PS, SM, Cer, HexCer, and TG, and a negative correlation with PC.

Marked increases in molar percentages of several species in PC class were seen after Peat 2 compared with WS (Fig. 3A). The increases of lipid species that contain 16:0 acyl chain (for example PC 32:0) after Peat 2 was an especially interesting finding. The Peat 2 samples also showed the lowest BALF cell count and neutrophil percentage, indicating resolution of the adverse airway reaction that was evident after WS. Thus, it appears that PC 32:0 is a feature of healthy airway cells. Previously, it was suggested that phospholipids are largely responsible for establishing the hydrophobic, barrier-providing surfaces of mucous membranes^58^. In animal models of gastrointestinal inflammation, exogenous PC has shown promise as an anti-inflammatory agent^58^. Since the relative concentration of PC 32:0 correlated negatively with BALF cell count but did not correlate with neutrophil or macrophage percentages, this response may be common for different cell types. In the PCA (Fig. 5B), the differences between the periods can be perceived in more detail as PC 32:0 (main molecular species 16:0_16:0) is especially enriched in the samples taken after Peat 2, whereas PC 36:4 (containing also 20:4n-6, arachidonic acid) is elevated after WS. In addition, the proportions of these two species have a strong negative correlation, making their ratio a promising marker of inflammation versus resolution, detected in this work already at subclinical stage.

The finding of increased PC species after Peat 2 compared with WS sampling may also be related to increased synthesis and secretion of this major phospholipid ingredient of surfactant. In adult mammals, including the horse, the phospholipid composition of surfactant is highly conserved, with PC covering 80–90% of the lipids in surfactant and PC 32:0 being the major phospholipid species^59–62^. Pulmonary surfactant is essential for life because of its actions in the lung, where it maintains the surface tension that prevents the alveoli from collapsing^61^. Alveolar type II epithelial cells synthesize and store surfactant intracellularly and secrete it via exocytosis. Thus, surfactant is found both intracellularly in alveolar type II cells and extracellularly. The secreted surfactant removes foreign material and sloughed cells from the airway peripheries. Alveolar macrophages may also contribute to the regulation of the amount and composition of surfactant lipids (e.g., by employing ABC cassette transporters)^51,62^. In human asthma and SEA, features related to surfactant (such as dysfunction, decreased amount, changes in phospholipid composition, and alterations in its distribution) contribute to disease mechanisms^32,58^. Albornoz et al.^22^ performed a metabolomics analysis on BALF samples retrieved from asthmatic horses and healthy horses during an episode of LPS inhalation-induced airway inflammation. BALF supernatant samples were subjected to metabolic analysis with gas chromatography-mass spectrometry (GC–MS). Decreases in palmitic (16:0), palmitoleic (16:1n-7), and oleic acids (18:1n-9) were found in both groups. This finding could be related to a change in the composition of surfactant, as these fatty acids are the main components of PC. Our result of increased PC levels (PC 32:0, PC 32:1) in BALF cells after Peat 2 may be explained by more surfactant produced by the cells as a compensatory means after a subclinical inflammatory episode during WS, as indicated by lipid markers.

The second highly interesting result of this study was the increased mol% of PI 38:4 after WS compared with both peat periods. This can be explained by the signalling role of arachidonic acid (20:4n-6) this specific PI molecule (PI 38:4) releases for production of lipid mediators (LMs)^63^. This finding suggests a pro-inflammatory state during WS, supported by the significant but minor changes in BALF cytology, such as increased cell counts and neutrophil percentages. This PI species is an important source of 20:4n-6 and is thus acting as a precursor for common inflammatory LMs, such as leukotrienes and prostaglandins. Cultured macrophages challenged by the innate immunity stimulant zymosan liberated especially efficiently 20:4n-6 from PI 38:4 for the synthesis of LTB_4_ and PGE_2_^64^. In our study, four additional polyunsaturated PI species were among the top 25 that increased in the BALF cells after WS (Fig. 5A). Leukotrienes, including LTB_4_, contribute to the pathogenesis of asthma^65,66^. In addition to the change in PI 38:4, the simultaneous reduction of PC 36:4 and PC 38:4 when transferred from WS to Peat 2 possibly indicates attenuating leukotriene and prostaglandin signalling, which uses 20:4n-6 of those PC species as the precursor^67^. However, the effects of PGE_2_ on lungs are both beneficial and deleterious depending on the target cells and receptors^68^.

There were changes between the bedding periods for several Cer species (Fig. 4B). Cer and other simple sphingolipids (SM and HexCer detected in this study) have certain lung-specific functions. They are not only minor structural components of surfactant and regulators of its synthesis and release, but also have roles in endothelial barrier maintenance and EV biogenesis^69^. There is considerable evidence that increased levels of Cer are involved in inflammatory airway diseases of laboratory animals and humans^38,39,70^. However, the role of sphingolipids in equine airway diseases has been investigated minimally. In our study on horse BALF cells, Cer and HexCer classes showed an increase after WS compared with Peat 1, but the change was not statistically significant. However, there was a significant decrease in Cer (*p* < 0.001) and HexCer (*p* < 0.001) levels after Peat 2 compared with Peat 1 or WS (Fig. 2). This may be related to the resolution of inflammation seen after Peat 2, also supported by the changed BALF cell counts and neutrophil percentages between these bedding periods.

In a recent study^32^, relative levels of the following sphingolipids were increased in surfactant samples from SEA cases: Cer 18:1;O2/16:0, Cer 18:1;O2/24:0, dihydroceramide Cer 18:0;O2/16:0, and hydroxy ceramide (OH-Cer) 18:1;O2/24:0;O. In our samples, the levels of hydroxy ceramides were very low and 18:0 dihydroceramides were not measured. There were both similarities and differences when the main findings of these surfactant results with those of our BALF cells were compared. In Christmann et al.^32^, Cer 18:1;O2/16:0 was elevated in SEA surfactant. In our cells, Cer 18:1;O2/16:0 was lower after the WS period compared with both peat periods, despite the WS appearing more pro-inflammatory in general, as seen in higher cell counts and higher PI 38:4. Interestingly, Cer 18:1;O2/24:0 was also elevated in SEA surfactant^32^, consistent with our results, where it was higher after WS compared to Peat 2. Similarly, HexCer 18:1;O2/24:0 was also higher after WS compared to Peat 2. In Christmann’s study^32^, HexCer 18:1;O2/24:0 in surfactant or plasma/serum remained unaltered between groups of horses with SEA, MEA, and healthy controls.

The differences in the results of Christmann’s study^32^ and ours are most likely due to two factors. First, the study populations were different. Our horses were healthy horses with mild adverse airway reaction with inflammatory lipid markers; the other study investigated horses with SEA. Moreover, in previous studies, surfactant- or cell-free BALF was used; in our study BALF cells were examined. This most likely leads to certain differences in the results, as Cers are present in surfactant in small amounts but are important cellular membrane elements. The sample type makes a marked difference when studying lipids. For instance, EVs, critical mediators of cell–cell communication, exhibited reduced amounts of Cer-phosphates and Cers in asthmatics compared with healthy controls^69^, although Cers have been high with asthmatics in many other studies using different kinds of samples^38,39,70^.

In addition to increased percentages of neutrophils, mast cells, or eosinophils^2,5^, increased total nucleated cell counts in BALF also indicate airway inflammation in horses^71,72^. Quantitative cell numbers of BALF are not commonly used as diagnostic determiners, as the variable dilutions of a BALF sample makes cell concentrations difficult to standardize. In our study, both the BALF cell count and the neutrophil percentages reflected the bedding material challenge, although the cytology results remained within normal reference range for all periods (neutrophils < 5%; < 400 cells/µL)^5^. Although neutrophil percentages mostly remained in the normal reference range, the wide range of normal variation and the ongoing discussion on the appropriate upper limits for BALF neutrophil percentages must not be forgotten. The design of the current study has the advantage of the horses acting as their own controls, and thus the cytology results can also be mirrored against their own results in addition to the generally accepted reference ranges. Therefore, although not significant regarding asthma diagnosis, these neutrophil increases may reflect the inflammatory status of the airways on an individual level. Some of the cytological findings correlated with the cell’s lipid class mol% values. This indicates that inflammatory reactions in the cellular lipid species composition can be detected even when cytology responses are weak.

There are some limitations in using BALF cytology as the gold standard for EA diagnosis, especially in MEA^8–17^. Although not all cases of MEA progress to a severe form of the disease, remodelling of bronchial-wall tissues has also been found from MEA cases^73^, highlighting the importance of early disease recognition. Asthma phenotypes in horses are poorly defined^74,75^ and lipidomics could offer a better understanding of the variation in disease pathophysiology. However, it should noted that lipid studies can address fatty acid, lipid, or LM profiles, and data comparisons between these approaches are challenging. In human medicine, the roles of numerous LMs in asthma pathogenesis have been recognized but are still poorly defined^40^. The disease heterogeneity is an obstacle to understanding the significance of the results from lipidomic studies^76^.

A limitation of this study is that the first BALF sampling was performed only after the horses had been stabled on peat bedding for one month, and “a day 0” sample representing circumstances before the first phase of the bedding challenge was not collected. The horses were on summer pasture before the first peat period. It is possible that a carryover effect caused by the possibly variable summer conditions was reflected in the results of the first sampling, as some horses may have been affected by summer pasture associated subtype of EA. Moreover, mere stabling of the horses can cause airway inflammation in healthy horses^1,77^. These points may explain the difference in results of Peat 1 and Peat 2 samplings, despite the bedding being the same for both periods.

There is not necessarily a single lipid molecule that would serve as a specific biomarker in disease. Rather, it may be possible to find common changes in the composition of the whole lipid profile at the lipid class or lipid species (or both) level, especially in the relations of proinflammatory and proresolving LMs^39^. In our PCA analysis (Fig. 5B), a compositional shift in the PC species profile separating Peat 2 and WS was found. With a myriad of significant changes, the results of lipidomic studies can be difficult to interpret. For example, in our study we detected 60 significant changes of lipid species; the only main classes that were unaltered were CE and PS at lipid-class level. Our study investigated the relations of different lipid classes and lipid species, and the results are presented as molar percentages. This resembles the situation with BALF cytology results, which are commonly presented as differentials of inflammatory cell populations. Relative proportions are used in both methods. This procedure normalizes the results to compositions that no longer reflect variation in the total cell counts and lipid concentrations among individual samples. At the same, this causes another bias; if for example PC amounts are increasing, this makes other subclass percentages appear smaller, even if they remained at their original level in terms of absolute concentration. Additionally, a technical limitation of our study method should be noted, as ether lipid species (PC alkyl species, PE alkenyl species) were not included in the analysis.

## Conclusions

The novel observation in this study was that the BALF-cell lipidome included numerous lipid species that indicated switching of the bedding even in clinically healthy horses with only mildly increased neutrophil percentages in BALF. This difference between the bedding periods was not observed with traditional cytology cut-offs. The lipid marker candidates included physiologically meaningful molecules, such as a PC species that serves as a major component of surfactant and a PI species releasing 20:4n-6 for production of LMs that modulate inflammation, bronchoconstriction, and other asthmatic responses.

### Supplementary Information

Supplementary Information 1.

Supplementary Information 2.

Supplementary Information 3.

Supplementary Information 4.

## Data Availability

The datasets used or analysed for the current study are available from the corresponding author on reasonable request.
