# Supplementary figures and images for: Lipid species profiling of bronchoalveolar lavage fluid cells of horses housed on two different bedding materials
Source: Sci Rep. 2023 Dec 8;13:21778. doi: 10.1038/s41598-023-49032-1 (PMC10709413; doi:10.1038/s41598-023-49032-1)

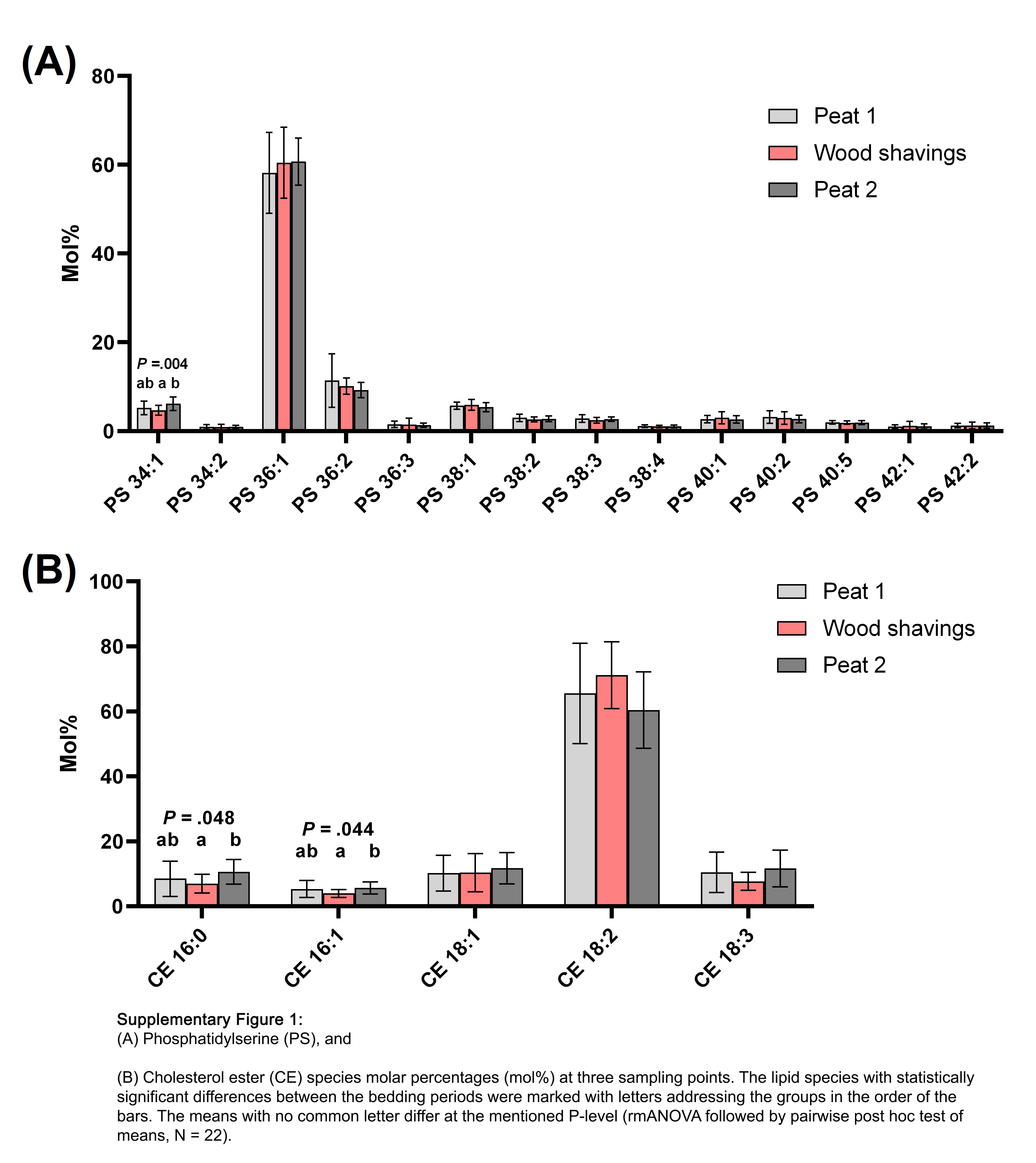

Supplement: Supplementary file 1 — Supplementary Information 1. [file 41598_2023_49032_MOESM1_ESM.jpeg]

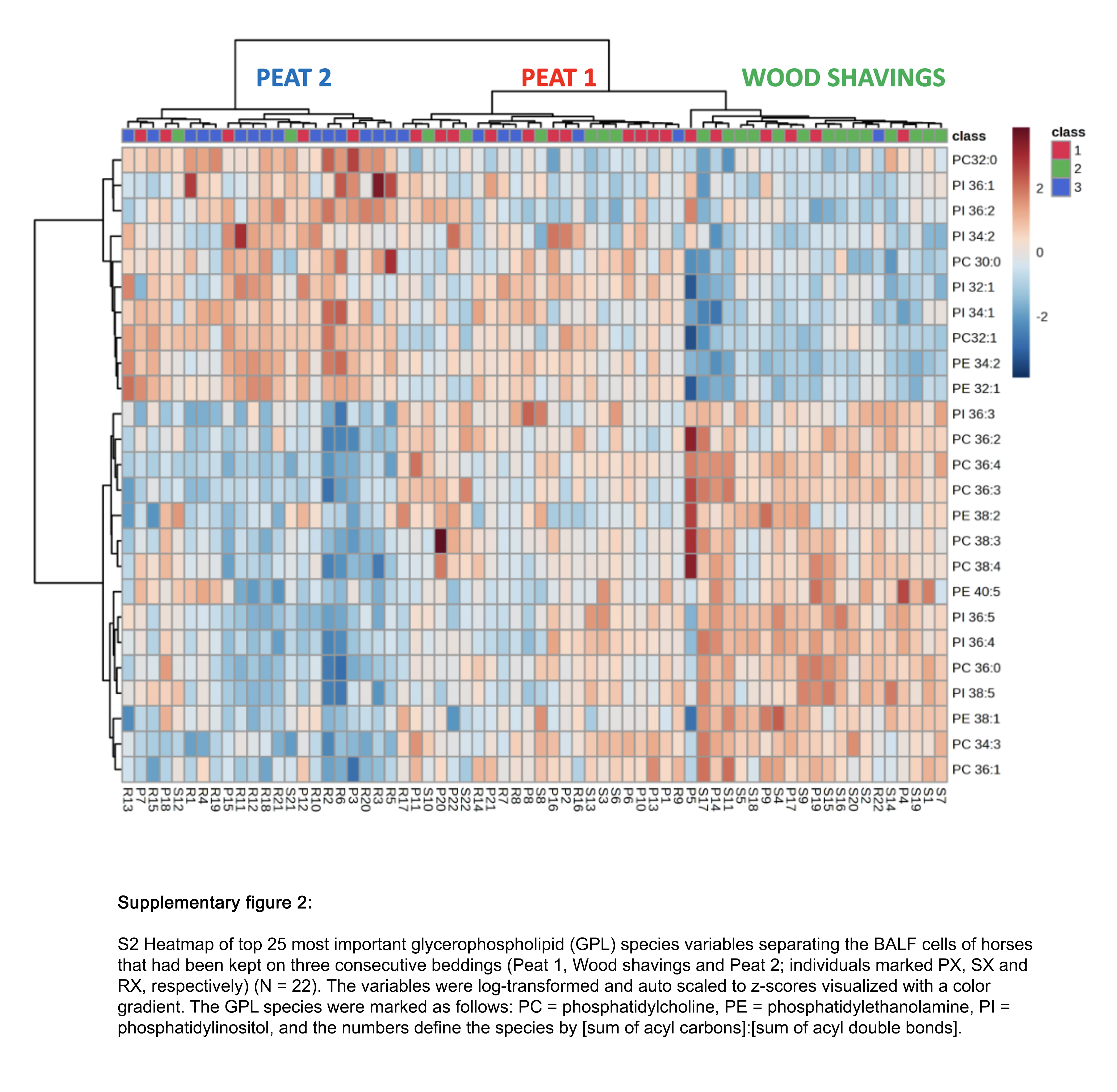

Supplement: Supplementary file 2 — Supplementary Information 2. [file 41598_2023_49032_MOESM2_ESM.jpeg]
